# Staying sane in the membrane: Neutral sphingomyelinase 2 as a master regulator of plasma membrane ceramide

**DOI:** 10.1016/j.jlr.2024.100737

**Published:** 2024-12-26

**Authors:** Zainuddin Quadri, Erhard Bieberich

**Affiliations:** Department of Physiology, University of Kentucky School of Medicine, Lexington, KY, USA

Ceramide, a key signaling sphingolipid in the plasma membrane, plays a pivotal role in fundamental cellular processes such as adhesion, polarity, and programmed cell death. The generation of plasma membrane ceramide is largely attributed to the activity of two types of sphingomyelinases: neutral sphingomyelinase 2 (nSMase2, Smpd3) and acid sphingomyelinase (aSMase, Smpd1). While many studies have explored ceramide generation following experimental activation of these enzymes, the mechanisms governing basal or steady-state ceramide levels have remained poorly understood. Using an innovative mass spectrometry approach developed in the Canals’ lab, the team has quantified the distinct contributions of nSMase2 and aSMase to plasma membrane ceramide homeostasis. Remarkably, their findings reveal that nSMase2 is the primary driver of ceramide regulation in the plasma membrane, establishing its critical role in maintaining ceramide homeostasis and signaling under physiological conditions.

## Ceramide: what you see is not always what you get

Ceramide is not a low-hanging fruit. While it is probably the most publicly recognized sphingolipid—owing to its starring role in the cosmetic industry - its true functionality remains far from being fully understood. This is not entirely surprising when comparing lipid biology to the technological advances in other research areas: While protein and nucleic acid research has seen enormous advancements, the study of sphingolipids only made its leap from thin-layer chromatography to precision mass spectrometry about three decades ago, a relatively recent shift compared to other fields. To make matters even more challenging, the research toolbox for sphingolipids is painfully sparse. Unlike proteins or nucleic acids, where fluorescent fusion proteins and labeled nucleotides revolutionized visualization and analysis in molecular biology, sphingolipids lack equivalent tools. When it comes to ceramide, only a few antibodies with sufficient specificity are available, and the use of fluorescently labeled ceramide binding proteins for detection is still in its early stages (1, 2, 3, 4). This scarcity of reliable tools makes the ability to quantify ceramide and its regulation at the plasma membrane a truly remarkable technological breakthrough.

**Figure 1:**
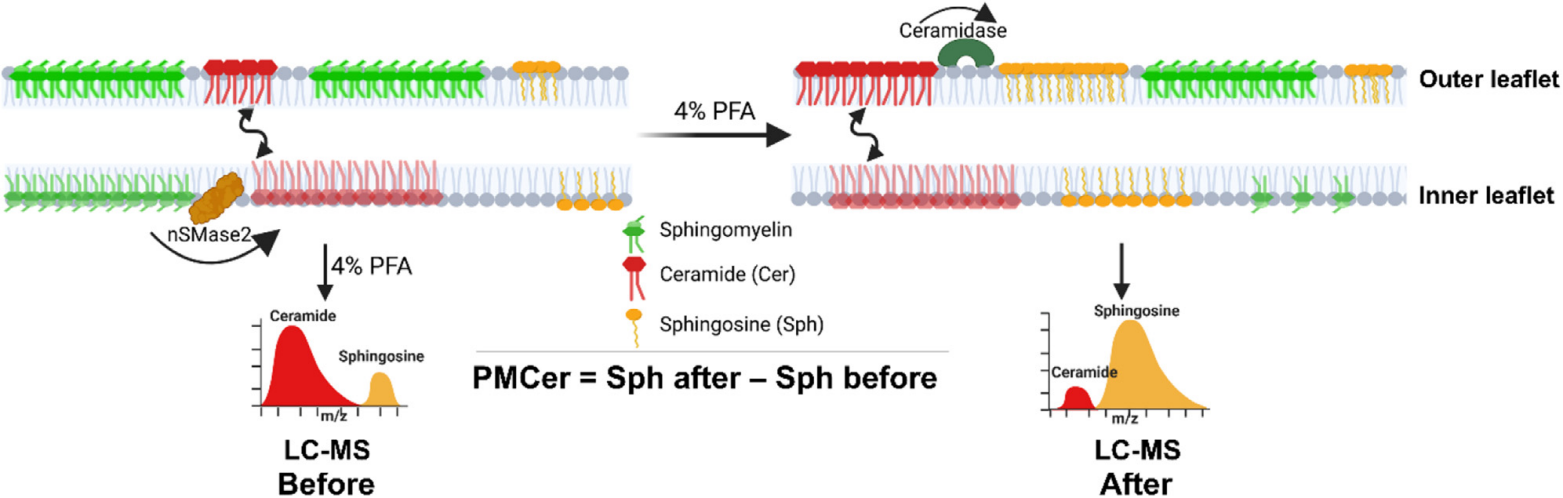
Schematics for plasma membrane ceramide (PMCer) quantification using LC-MS. nSMase2 converts sphingomyelin into ceramide which freely flips between the inner and the outer leaflet and can be quantified after fixation of cells with 4% p-formaldehyde (PFA). Plasma membrane ceramide (PMCer) is converted to sphingosine by recombinant bacterial ceramidase and then sphingosine quantified using liquid chromatography-mass spectrometry (LC-MS). The difference in sphingosine before and after treatment with ceramidase is equivalent to PMCer, the concentration of which is controlled by nSMase2.

The innovative mass spectrometric approach to understand the regulation of plasma membrane ceramide builds upon the initial method for quantifying ceramide in the plasma membrane developed by the Canals’ group (5, 6). Previously, mass spectrometric analysis required separation of cellular organelles and compartments, a process that was often prone to cross-contamination of cell fractions. To address this limitation, the Canals’ group fixed cells with p-formaldehyde and used recombinant bacterial (*Pseudomonas aeruginosa*) ceramidase (pCDase) to convert outer leaflet ceramide into sphingosine (6). Fixation was necessary to prevent further metabolization of sphingosine to sphingosine-1-phosphate, which rapidly occurred when cells were alive. Importantly, pCDase could not enter the fixed cells ensuring that intracellular ceramide remained unaffected. However, ceramide could freely flip between the inner and outer leaflets of the plasma membrane, even in fixed cells, resulting in the complete conversion of plasma membrane ceramide into sphingosine (Fig. 1). The sphingosine levels were then quantified via mass spectrometry, and the plasma membrane ceramide content was calculated as the difference in sphingosine levels before and after pCDase treatment. Since the difference was only contributed by ceramide hydrolyzed in the plasma membrane, the group was able to precisely determine the proportion of plasma membrane ceramide.

The findings were remarkable. Previous studies using anti-ceramide antibodies or fluorescently labeled ceramide binding proteins to visualize plasma membrane ceramide suggested a significant proportion of ceramide residing in the plasma membrane (1, 2, 7). However, the mass spectrometric method developed by the Canals’ lab revealed an astonishingly low concentration—less than 1% of total ceramide—in the plasma membrane of resting HeLa cells (5). Moreover, applying this method to various cell types revealed that plasma membrane ceramide levels do not correlate with total ceramide levels, highlighting the need for caution when making functional predictions based solely on total ceramide levels (6). This discovery is pivotal for understanding the regulation and function of ceramide for cell signaling pathways controlling biological processes ranging from cell adhesion to programmed cell death. It is also important to note that under stress conditions such as incubation with doxorubicin, a chemotherapeutic agent known to elevate ceramide to induce cancer cell death, increased the level of plasma membrane by up to 30-fold (5). This striking observation highlights the dynamic nature of ceramide regulation and will be revisited when discussing the role of sphingomyelinases in the generation of plasma membrane ceramide.

## Plasma membrane ceramide: better on a raft than swimming in an ocean

The discrepancy between what you appear to “see” through antibody labeling and quantify through mass spectrometry may not be as surprising as it seems. Biophysical studies have long demonstrated that lipids are not homogeneously distributed across the plasma membrane as originally proposed in the Singer-Nicolson model but are instead organized into concentrated clusters known as microdomains or lipid rafts. To date, there is substantial experimental evidence supporting the existence of lipid rafts in fixed cells and in fractionated membranes. However, their conclusive detection or visualization in living cells remains elusive. This challenge arises from the inherent technical limitations of directly visualizing lipids using fluorescent methods. Fluorophores, while useful when attached to proteins and oligonucleotides, alter the biophysical properties of lipids, making them unreliable for detecting rafts in their native state within living cells. Despite this limitation, numerous studies have successfully predicted the outcomes of biological processes by hypothesizing that lipid rafts, which organize receptor-mediated cell signaling pathways, cytoskeletal anchor sites, or other membrane-controlled mechanisms play critical roles in sphingolipid function within the plasma membrane. Although direct observation of these processes remains challenging, the predictive power of this model underscores its relevance in understanding ceramide function.

Ceramide, sphingomyelin, and cholesterol are thought to form the classical type of lipid rafts. However, an increase in ceramide levels through activation of sphingomyelinase(s) can displace cholesterol, leading to the formation of ceramide-rich platforms (CRPs) (8). Super resolution microscopy using anti-ceramide antibodies has revealed that CRPs are relatively small, with a size of 75 nm, corresponding to >20 ceramide molecules (9). It is unlikely that the binding epitope for ceramide antibodies is confined solely to the small polar head group accessible at the plasma membrane. Instead, these antibodies likely recognize ceramide clusters within a specific lipid environment. Consequently, some ceramide antibodies may strongly bind to CRPs, regardless of whether they are dispersed throughout the membrane, as seen in many cancer cells, or concentrated in specific regions, such as the polarized membrane protrusions (eg, cilia) of primary cultured cells or differentiated stem cells (10, 11). Moreover, lipids cannot be fixed by paraformaldehyde, and ceramide may flip between the inner and outer leaflets of the plasma membrane. However, lipids in rafts may become laterally confined when raft proteins are cross-linked during fixation. These factors may contribute to the disconnect between antibody labeling and the actual ceramide levels in the plasma membrane, underscoring the critical need to understand how ceramide is generated and regulated in a site-specific manner.

### nSMase2 and ceramide: a new master of the membrane

The concentration of ceramide in the plasma membrane mainly relies on the activity of two enzymes: nSMase2 at the inner (cytoplasmic) leaflet and aSMase at the outer (exoplasmic) leaflet or in lysosomes. Previous studies have largely attributed the regulation of ceramide levels in CRPs to aSMase (7). This is reasonable given that 70%–80% of cellular sphingomyelin is estimated to reside in the outer leaflet (12). However, the conclusion that plasma membrane ceramide is regulated by aSMase is largely drawn from experiments performed under stress-inducing conditions, such as cytokine activation and oxidative stress. In contrast, our laboratory has provided substantial evidence that nSMase2 plays a critical role in the site-specific generation of polarized membrane protrusions, including cilia and filopodia (11, 13). This suggests that nSMase2 is more intricately involved in fine-tuned cellular processes linked to cytoskeletal organization, whereas aSMase-mediated ceramide generation may predominantly be a response to acute cellular stress or external stimuli and insults. The innovative technology developed by the Canals’ group now enables precise and quantitative assessment of how nSMase2 and aSMase contribute to plasma membrane ceramide dynamics. This approach provides critical insights into the distinct roles of these enzymes in maintaining steady-state conditions and responding to acute challenges.

To identify the enzyme critically responsible for regulating plasma membrane ceramide, the Canals’ team overexpressed several enzymes known to affect ceramide levels, including aSMase, nSMase2, glucocerebrosidase, and sphingomyelin synthase 2. While aSMase, nSMase2, and glucocerebrosidase all increased total ceramide levels, only nSMase2 significantly elevated ceramide in the plasma membrane (6). Other neutral sphingomyelinases, such as nSMase1 (Smpd2) and nSMase3 (Smpd4), did not affect plasma membrane ceramide levels. Remarkably, overexpression of sphingomyelin synthase 2, a plasma membrane-bound enzyme that converts ceramide to sphingomyelin, dramatically increased plasma membrane sphingomyelin as expected but did not alter plasma membrane ceramide. Similarly, overexpression of acid ceramidase and neutral ceramidase, enzymes known to hydrolyze ceramide at the plasma membrane, reduced total ceramide and increased sphingosine levels but had no significant effect on plasma membrane ceramide. These results clearly show that the level of plasma membrane ceramide is tightly regulated by nSMase2, which replenishes the steady-state ceramide level as needed. Consistent with this role, the knockdown of nSMase2 led to a significant reduction in plasma membrane ceramide, further highlighting its critical function as the master regulator of ceramide homeostasis at the plasma membrane.

### The plasma membrane: a well-regulated dynamic border under steady-state and stress

**Figure 2:**
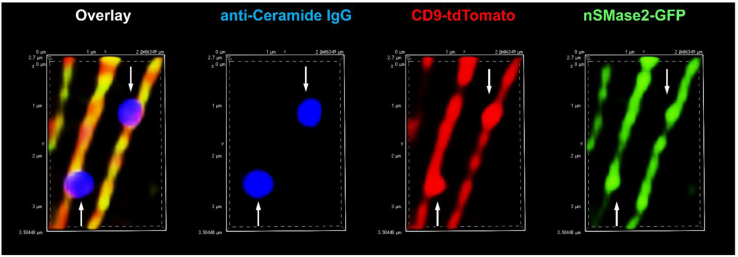
Visualization of ceramide using anti-ceramide antibody in HeLa cells. HeLa cells overexpressing nSMase2-GFP and CD9-tdTomato were immunolabeled with anti-ceramide rabbit IgG as described in (13). Arrows point at presumptive extracellular vesicles budding off filopodia.

Textbook schematics often misrepresent the plasma membrane as a static barrier or stretchable balloon. In reality, any membrane protrusion or invagination increases surface area, requiring dynamic vesicle-mediated transport and the tightly regulated activation of enzymes that adjust membrane lipids, such as ceramide. The recent study by the Canals’ lab identifies nSMase2 as the key enzyme regulating the ceramide concentration in the plasma membrane under both steady-state conditions and varying cell densities (6). Cell density is particularly intriguing as it links membrane dynamics with the cytoskeleton and cell adhesion. The observed increase in nSMase2 activity and ceramide levels at confluence suggests its role in setting boundary conditions for membrane extension, whether as intercellular borders or in structures like cilia and filopodia. These findings align with our own work on filopodia generation and extracellular vesicle (EV) secretion in HeLa cells, where inhibiting or knocking down nSMase2 reduced filopodia formation and EV secretion (13). In contrast, inhibition of aSMase decreased EV secretion without affecting filopodia formation, highlighting the distinct and critical role of nSMase2 and ceramide in plasma membrane remodeling. The low baseline concentration of ceramide in the plasma membrane, as reported by the Canals’ lab, is reflected in immunolabeling experiments using a ceramide antibody developed in our laboratory (3). In HeLa cells overexpressing nSMase2-GFP and the EV marker CD9-tdTomato, ceramide-enriched EVs bud from the membrane, while most of the plasma membrane remains unlabeled (Fig. 2) (13). This site-specific ceramide generation is consistent with the concept of CRPs and highlights the central role of nSMase2 in organizing ceramide domains.

However, what happens when cells are challenged by stress? When HeLa cells were exposed to oxidative stress, we observed increased filopodia formation and EV secretion (13). Consistent with the effect of inhibitors on non-stressed cells, inhibiting either nSMase2 or aSMase blocked EV secretion, but only nSMase2 inhibition reduced filopodia formation (13). Notably, aSMase seems to complement the activity of nSMase2 by promoting EV secretion, likely originating from filopodia. These findings support and extend the conclusions of the Canals’ lab. Under steady-state conditions, nSMase2-mediated ceramide production drives filopodia formation. Under oxidative stress, aSMase further increases ceramide levels, promoting EV secretion. Importantly, this process is self-regulating: ceramide-rich EVs (termed CREVs) are secreted, reducing membrane ceramide and reestablishing equilibrium.

In summary, the combined evidence from the Canals’ lab and our work suggests that nSMase2 plays a critical role in maintaining ceramide homeostasis under steady-state conditions, while aSMase predominantly drives ceramide generation during stress response. Together, these enzymes coordinate a well-regulated and dynamic process that integrates plasma membrane remodeling, EV secretion, and vesicle transport.

## Data availability

All data generated or analyzed during this study are included in this published article and its supplementary information files.

## Conflict of interests

The authors declare that they have no conflicts of interest with the contents of this article.
